# Fish and amphibians as potential reservoirs of *Mycobacterium ulcerans*, the causative agent of Buruli ulcer disease

**DOI:** 10.3402/iee.v3i0.19946

**Published:** 2013-02-22

**Authors:** Sarah J. Willson, Michael G. Kaufman, Richard W. Merritt, Heather R. Williamson, David M. Malakauskas, Mark Eric Benbow

**Affiliations:** 1 Department of Entomology, Michigan State University, East Lansing, MI, United States; 2 Department of Microbiology, University of Tennessee, Knoxville, TN, United States; 3 Department of Biology, University of Dayton, Dayton, OH, United States

**Keywords:** mycolactone, enoyl reductase, Ghana, Africa, VNTR, Mycobacterium liflandii, inhibition, ecology

## Abstract

**Background:**

Buruli ulcer is a skin disease often associated with proximity to certain water bodies in Africa. Much remains unknown about the reservoir and transmission of this disease. Previous studies have suggested that fish may concentrate *Mycobacterium ulcerans*, the etiological agent of the disease, in their gills and intestines and serve as passive reservoirs of the bacterium. The objective of this study was to test the hypothesis that fish and amphibians serve as natural reservoirs of *M. ulcerans* or other closely related mycolactone-producing mycobacteria.

**Methods:**

Polymerase chain reaction targeting the enoyl reductase (ER) domain present in mlsA, which is required for mycolactone production, was used to screen water, fish, and amphibians from water bodies in Ghana for the presence of mycolactone-producing mycobacteria, and positive specimens were subjected to variable number tandem repeat (VNTR) typing.

**Results:**

The use of VNTR typing revealed the presence of *Mycobacterium liflandii* in a tadpole and a fish, and *M. ulcerans* in an adult frog. Similarity percentage analysis (SIMPER) showed that the predatory cichlid *Hemichromis bimaculatus* was associated with ER-positive water bodies. No amphibian species or fish-feeding guild served as a reliable indicator of the presence of mycolactone-producing mycobacteria in a water body, and there was no significant difference between fish and amphibian positivity rates (*P*-value=0.106). There was a significant difference between water bodies in the total number of ER-positive specimens (*P*-value=0.0164).

**Conclusions:**

Although IS*2404*-positive tadpoles and fish have been reported, this is the first VNTR confirmation of *M. ulcerans* or *M. liflandii* in wild amphibian and fish populations in West Africa. Results from this study suggest that amphibians should be carefully examined as potential reservoirs for *M. ulcerans* in West Africa, and that *H. bimaculatus* may be useful as an indicator of habitats likely to support mycolactone-producing mycobacteria.

*Mycobacterium ulcerans* MacCallum is the causative agent of Buruli ulcer disease (1). In humans, Buruli ulcer disease manifests first as a small nodule and can progress to extensive skin ulcerations. This disease tends to occur in tropical and subtropical areas and is especially prevalent in the West African countries of Benin, Ivory Coast, and Ghana (2). Local outbreaks have been linked to exposure to certain water bodies, but the reservoir(s) and the method of transmission of *M. ulcerans* have not been determined (3–9). Possible hosts of *M. ulcerans* include predaceous aquatic insects (10, 11), fish (12), snails (13), possums (14), mosquitoes (15), and turtles (16). In aquatic environments, *M. ulcerans* may be concentrated by small filtering organisms, such as filter-feeding insects, which are then preyed upon by larger organisms, causing further concentration of the bacterium (17, 18). The recent discovery of mycolactone-producing mycobacteria in frogs (19) also has raised the question of whether or not *M. ulcerans* might occur on or in frogs. Many tadpoles feed by filtering or scraping small algal particles and could conceivably acquire and concentrate *M. ulcerans* during this process. Associations of *M. ulcerans* with amphibians would demonstrate a terrestrial ecological link similar to the link between *M. ulcerans* and possums, which has been observed in Australia (14).

*M. ulcerans* is a member of the *Mycobacterium marinum* complex of temperature-sensitive mycobacterial pathogens of aquatic species (5). It is believed to share a common ancestor with *M*. *marinum*, a widely disseminated pathogen of fish (20, 21). *M. ulcerans* has acquired a virulence plasmid, pMUM001, which allows production of mycolactone (22). Mycolactone is a toxin that appears to play an important role in allowing *M. ulcerans* to use specific hosts (23). Plasmids encoding mycolactone have only been identified in pathogenic members of the *M. marinum* complex, which have been found to infect fish (24, 25) and frogs. *Mycobacterium liflandii*, a mycolactone-producing mycobacterium closely related to *M. ulcerans*, is a cause of lethal infection in West African clawed frogs used in Europe and the United States in research laboratories (19). Though currently given separate species names, it has been argued that all mycolactone-producing mycobacteria are different strains of a single species (21, 26).

Previous studies suggested that fish may concentrate *M. ulcerans* in their gills and intestines and then serve as passive reservoirs of the bacterium (12, 27, 28). In these studies, the gills and intestines of several fish were found to be positive for IS*2404*, an insertion sequence previously thought to be specific to *M. ulcerans*, but now known to occur in other mycobacteria (29). Whether these reported positive IS*2404* associations resulted from the presence of mycobacteria other than *M. ulcerans* or from *M. ulcerans* cannot be determined based on the use of a single target sequence. Though mycobacteriosis in fish can involve multiple organs, it is most commonly detected as a chronic granulomatous infection of the kidney and liver (30).

Since the first reports of IS*2404*-positive fish, more specific polymerase chain reaction (PCR) methods have been developed to detect *M. ulcerans* in environmental samples. For example, enoyl reductase (ER) PCR targets the ER domain of *mlsA*, the polyketide synthase that encodes the lactone core of mycolactone (31). This target is present in four copies, making ER-PCR less sensitive but more specific than IS*2404* PCR (32). An ER-positive sample provides strong evidence for the presence of mycolactone-producing mycobacteria in the *M. marinum* complex. More definitive evidence of *M. ulcerans* can be provided by variable number tandem repeat (VNTR) DNA typing, which makes it possible to distinguish between *M. ulcerans* and other mycolactone-producing mycobacteria. VNTR loci occur in varying numbers, with some occurring only once in the genome making this the least sensitive, though highly specific, PCR detection method (33). Positive assay results are dependent on currently known VNTR profiles, and it is likely that additional profiles exist.

The present study was designed to expand on earlier studies by screening water samples and multiple species of fish and amphibians from multiple water bodies in Buruli ulcer endemic and non-endemic areas to test the hypothesis that fish and amphibians serve as natural reservoirs of *M. ulcerans*. For initial collections, endemicity classification was based on district-level human case data. The study was designed to determine the distribution of *M. ulcerans* within specific taxa or feeding guilds to determine whether some species or feeding groups were more likely to harbor the pathogen than others. It was predicted that certain fish and amphibian taxa could be used as indicator species for the presence of environmental conditions favorable to *M. ulcerans*. In addition, because fish and amphibians are mobile (increasing the chance of contact with the bacterium), and provide a stable environment for the bacterium, PCR testing of fish and amphibians might allow for more consistent detection of *M. ulcerans* in a water body than other environmental sampling methods such as water filters. To accomplish these goals, a survey of fish and amphibian taxa was conducted in 31 sites in both *M. ulcerans* endemic and *M. ulcerans* non-endemic locations in Ghana. Specimens were collected from *M*. *ulcerans* endemic areas for subsequent PCR screening for mycolactone-producing mycobacteria, and positivity rates were compared for internal organs versus external swabs, between different taxa, and between feeding guilds.

## Methods

### Study location

**Fig. 1:**
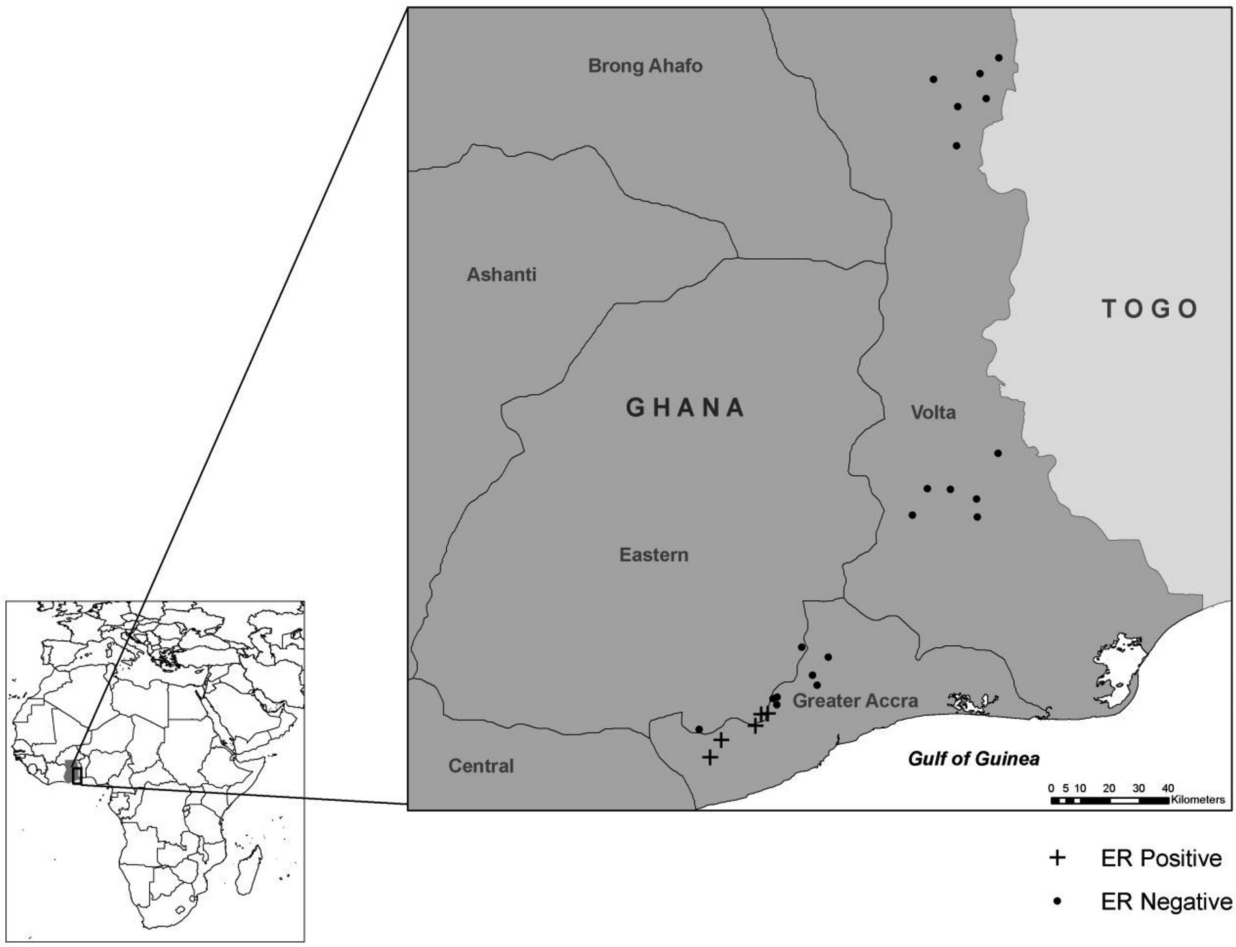
Sampling locations from 2008. Presence of mycolactone-producing mycobacteria was determined by subjecting water filter samples to PCR, targeting the enoyl reductase (ER) domain present in the plasmid required for mycolactone production.

**Fig. 2:**
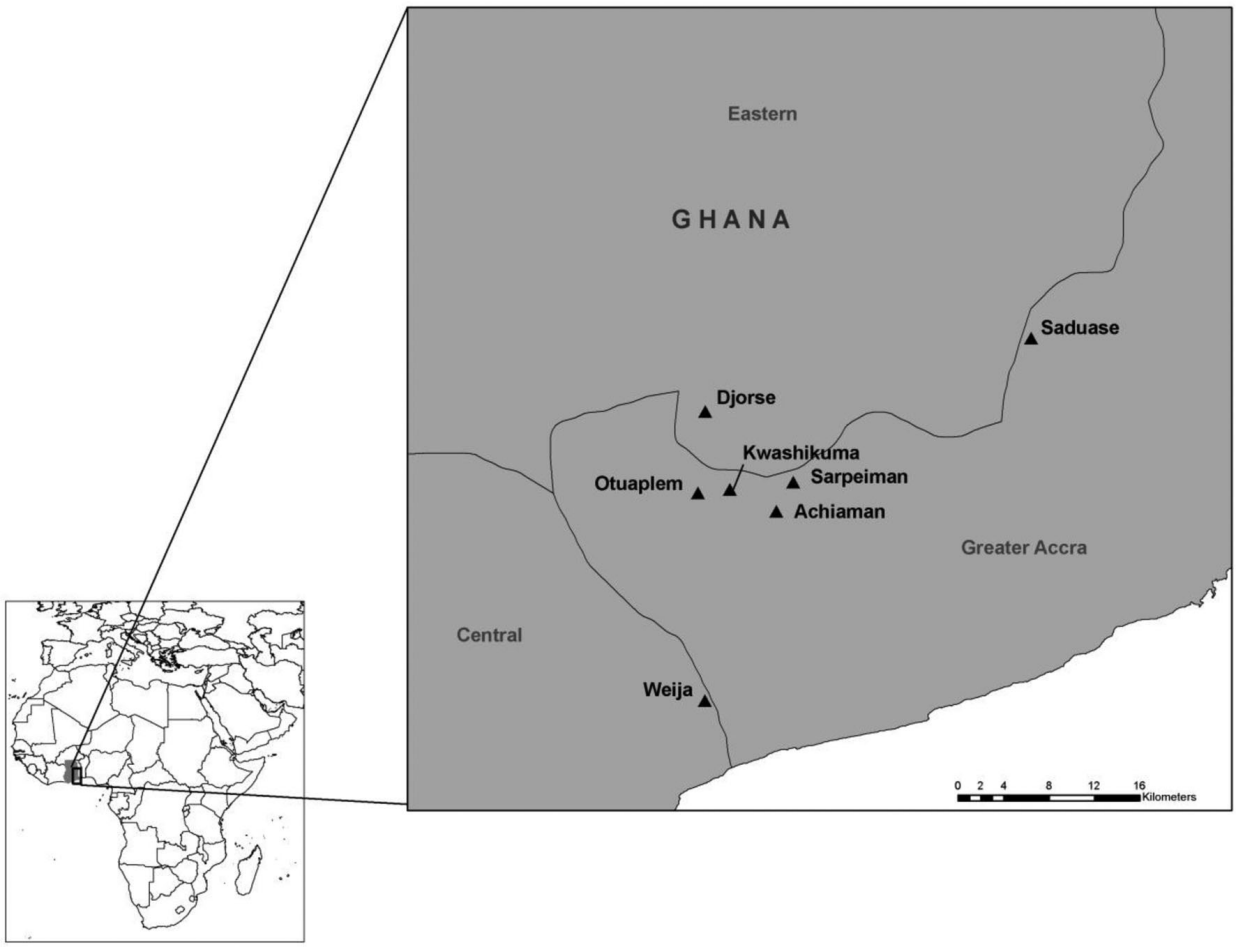
Sampling locations from 2009. Mycolactone-producing mycobacteria were detected at all locations as determined by subjecting fish and amphibian tissue samples to PCR, targeting the enoyl reductase (ER) domain present in the plasmid required for mycolactone production.

This study was carried out as part of a larger research project on the ecology of *M. ulcerans* in West Africa. Samples were collected in July 2008 from 25 sites in Ghana. Sites were located along a gradient from the Volta Region, where no *M. ulcerans* or mycolactone-producing mycobacteria have been detected, to the Greater Accra area, where *M. ulcerans* and other mycolactone-producing mycobacteria have been detected. Buruli ulcer disease is largely absent from the Volta Region, and it is endemic to the Greater Accra area (Fig. 1). A second sampling season occurred in August 2009, in which six sites were sampled in the Ga West District of Accra, Ghana, where *M. ulcerans* is considered to be endemic, and one site was sampled in the Tema district of Accra, Ghana, where *M. ulcerans* is not endemic (31, 34) (Fig. 2). It is unclear whether there is a relationship between seasonality and Buruli ulcer disease occurrence in West Africa (5); however, both sampling efforts in the current study were conducted in late July/early August to minimize any potential seasonal impacts.

### Collection and preservation of specimens

Sample collection procedures varied between 2008 and 2009 due to differing objectives for each season. In 2008, the primary objective was to conduct an aquatic community analysis to determine whether certain fish or amphibian species were associated with ER-positive water bodies. For the 2008 season, water samples were collected for DNA processing and fish and amphibians were collected for identification but not for DNA analysis. In 2009, the primary objective was to determine whether fish and amphibians were potential reservoirs of *M. ulcerans* or other mycolactone-producing mycobacteria. For the 2009 season, fish and amphibians were collected for DNA processing. External swabs were taken from a subsample of fish and amphibians in 2009 to clarify whether positive results were due to external contamination. Water samples were not processed for the 2009 season because of the high expense of processing and because most samples were collected from sites that could reasonably be expected to be ER-positive due to either human endemicity data or previously collected ER-positive samples. DNA testing rather than bacterial cultures was used because *M. ulcerans* has a slow growth rate, making it extremely difficult to culture from environmental samples where other faster-growing contaminating microorganisms are present (5).

Three fish- and amphibian-sampling techniques were employed at each site to maximize the variety of taxa captured. Three collapsible live bait traps (Promar model TR-501, Gardena, CA) were deployed at each site and baited with commercial catfish dough bait (Berkley, Spirit Lake, IA), and the traps were set as far apart as possible around the site. Traps were submerged for approximately one hour at each site. In addition, a seine (Cabela's Inc., Sidney, NE) was used when the sites were wadeable and without abundant aquatic vegetation. A D-net (Bioquip Products, Rancho Dominguez, CA) was used to sample marginal areas and within aquatic plant beds. Approximately 1 hour of combined seining and D-net sampling was performed at each site. When adult frogs were observed at a site, they were captured with D-nets. Up to 30 individuals of each fish and amphibian taxon present at the site were captured, and excess individuals were released. Two exceptions to these methods occurred in 2009. A higher number of specimens were collected from Kwashikuma because a pond as well as an adjacent marsh area was sampled, and at Lake Weija, live fish were purchased from local fishermen and no amphibian collections were attempted. Fish and tadpoles were euthanized with CO_2_, and adult frogs were euthanized with benzocaine hydrochloride (IACUC AUF # 06/08-090-00). Buckets used to hold captured fish and amphibians were cleaned and treated with RNase AWAY (Molecular Bioproducts, Inc., San Diego, CA) between sites.

In 2009, external swab samples were taken from 25 randomly chosen fish and amphibian specimens collected from Kwashikuma. This was done to determine the likelihood of positive DNA results being caused by bacteria adhering to the external surface of a specimen. Immediately following euthanasia of each specimen, the entire external surface of each specimen was rubbed with a sterile swab, which was then preserved in ethanol. An additional two swab samples were taken from tadpoles captured at Otuaplem that showed outward signs of potential infection.

Water was collected and filtered at all sites in 2008 to test for the presence of *M. ulcerans* and other mycolactone-producing mycobacteria. 20 water filters were analyzed per site, 10 from water samples collected in open water and 10 from water collected near aquatic vegetation. Samples were initially filtered through a 1.6 µm fiberglass filter to remove larger debris, and then filtered through a 0.2 µm nitrocellulose filter, as described in previously published methods (31).

In 2008, all tadpoles were preserved in 10% formalin and all fish were preserved in 95% ethanol, and all organisms from a single site were preserved together. For 2009, half of all specimens (fish and tadpoles) were preserved in 10% formalin and half were preserved in 95% ethanol. Formalin was used to preserve voucher specimens for taxonomic identification. Ethanol was used for specimens intended for DNA processing. Specimens in ethanol were either preserved individually or pooled into groups of three depending on size.

Fish and amphibians were identified to the highest taxonomic resolution possible using appropriate keys (35–37). For DNA analysis, specimens were dissected and the intestines and kidneys were removed and stored in 95% ethanol. The kidney was chosen because though mycobacteriosis in fish can potentially involve any of the organs, it most commonly involves the kidney and liver (30). A thin membrane separates the kidney from the other internal organs, minimizing chance of cross-contamination during dissection. While gills were tested in previous fish studies, they were not included in this study because it would be difficult to rule out incidental contamination of gills. With fish artificially infected with *M. marinum* and *M. peregrinum*, mycobacteria were subsequently recovered from internal organs of infected fish but never from the gills (38). The intestine was chosen because experimental exposure of *Danio rerio* to *M. marinum* and *M. peregrinum* showed that these mycobacteria are primarily acquired through the intestine before disseminating to other organs (30). Intestine results were used to show whether fish and amphibians ingested mycolactone-producing mycobacteria, while kidney results were used to ascertain if ingested mycobacteria produced an internal infection.

Instruments were cleaned between specimens using 95% ethanol followed by application of RNase AWAY.

### DNA extraction

Samples from 2009 preserved in 95% ethanol were screened for ER-positive DNA. All DNA extractions and PCR procedures were performed in a hood, under sterile conditions. Negative controls were used throughout the process to ensure sterility and assess possible contamination. Cultured *M. ulcerans* cells preserved in 95% ethanol were used as a positive control to ensure that procedures were successful. For small samples (less than or equal to 20 mg wet mass), the entire sample was used for DNA extraction. For large samples, homogenate containing approximately 20 mg of tissue was used for DNA extraction. Samples were centrifuged to remove ethanol before DNA extraction. The remaining pellet was rinsed with TE, centrifuged, and the supernatant was removed.

Extraction was first attempted using the one tube method designed for extraction of *M. ulcerans* in aquatic insects, mollusks, and fish (12). However, satisfactory detection was not achieved with this method. DNA was then extracted using the Käser method, which is optimized for environmental mycobacteria (39). However, in the final step, DNA was re-suspended in 25 µl TE buffer instead of the 100 µl water used in the Kaser method. To test this method, serial dilutions were made from a stock sample of *M. ulcerans* cells in 95% ethanol that contained 10^7^ CFUs/ml. Aggregates of bacteria were broken apart by passing the bacterial suspension through a 25-gauge needle 10 times, and eight 1:10 serial dilutions were made (31). To determine whether sensitivity was changed by the addition of fish tissue, the internal organs of a commercially purchased goldfish were dissected and processed using the previously mentioned techniques. Also, 180 µl of 0.1 mg/µl (wet mass) tissue homogenate was spiked with 20 µl of each of the previously made dilutions before being subjected to the Käser extraction procedure (39) and ER-PCR.

Extractions were performed in groups of 18 samples, and each group included a negative and a positive control. Extracted DNA from a subset of 27 intestine samples and 23 kidney samples was spiked with *M. ulcerans* DNA prior to PCR analysis to determine the level of PCR inhibition. Due to the presence of inhibitors, it was necessary to further purify the extracted intestinal DNA according to manufacturer's instructions using PowerClean DNA Clean-Up Kits (MO BIO Laboratories, Inc., Carlsbad, CA), eluting with 50 µl of the provided elution solution. Negative and positive controls were also cleaned using this procedure. Because the clean-up of the intestine samples resulted in more dilute DNA than that of the kidney samples, 8 µl of intestine sample DNA versus 4 µl of kidney sample DNA was used per 25 µl PCR mixture.

### PCR and gel electrophoresis

PCR was performed targeting the ER domain of the plasmid responsible for mycolactone production. The 25 µl PCR cocktails contained 2 µl of each primer (10 µM), 12.5 µl FailSafe 2×PCR buffer (EPICENTRE Biotechnologies, Madison, WI), 0.5 µl of Go Taq polymerase enzyme (Promega, Madison, WI), and either 4 µl of template DNA and 4 µl of PCR water (for kidney samples) or 8 µl of template DNA (for intestine samples). PCRs were run using previously described primers and cycling conditions (31). Samples were loaded along with positive and negative controls onto 0.8% TBE agarose gels stained with ethidium bromide, and fragment sizes were compared to a 1 Kb DNA ladder (Invitrogen, Carlsbad, CA). All ER-positive samples were then sent to the University of Tennessee for VNTR analysis as previously described (31).

### Data analyses

We sought to determine if there was a difference in fish or amphibian communities at sites that tested positive or negative for the presence of mycolactone-producing mycobacteria. We also sought to determine which taxa contributed to these differences. A Jaccard similarity matrix was constructed separately for both fish and amphibian communities using data collected in 2008, and combined data from 2008 and 2009. A Jaccard similarity matrix allows the comparison of species diversity between each pair of sites by analyzing the presence and absence of taxa (40). Separate non-metric multidimensional scaling (NMS) ordinations were performed on fish and amphibian community ranks obtained from Jaccard matrices using ER positivity as a grouping variable. NMS ordinations allow for visualization of relative similarity between sites; sites that appear closer together in the ordination are more similar (41). Analyses of similarity (ANOSIM) were also performed. ANOSIM is an iterative approach that compares within group similarity ranks to between group similarity ranks to determine if there is a statistical difference between groups (42). ANOSIMs were performed using 10,000 permutations to determine if there were differences between ER-positive and ER-negative fish or amphibian communities. A similarity percentage analysis (SIMPER) was also conducted to determine which taxa were most responsible for the differences between ER-positive and ER-negative sites (42). The above statistical tests were performed using PAST 2.16. Site ER classification for data analyses was based upon water filter results for sites visited in 2008, and on fish and amphibian ER results for sites visited in 2009. Separate Fisher's exact tests were performed to test for differences in positivity between sites, fish and amphibians, and fish-feeding guilds (R Development Core Team 2012). All results were considered to be significant at α=0.05, and all *P*-values were corrected for multiple tests.

## Results

### Fish and amphibian community analysis

**Fig. 3:**
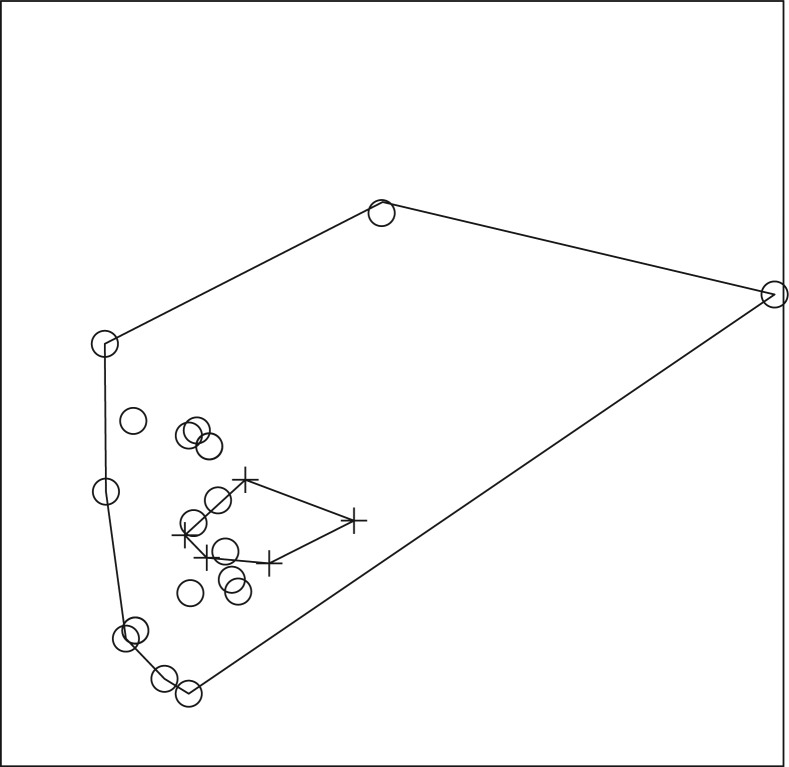
NMS ordination plot with three dimensions represented on two axes, including convex hulls, representing solution space of fish communities, using species presence or absence data from 2008 in relation to enoyl reductase (ER) domain positivity. Crosses represent ER-positive sites and circles represent ER-negative sites. Stress: 0.4665. Sites that cluster closest together in the ordination have the most similar community structure. Considerable overlap of sites demonstrates a lack of significant difference (ANOSIM; *P*-value=0.672) in fish community structure in ER-positive versus ER-negative sites in 2008.

**Fig. 4:**
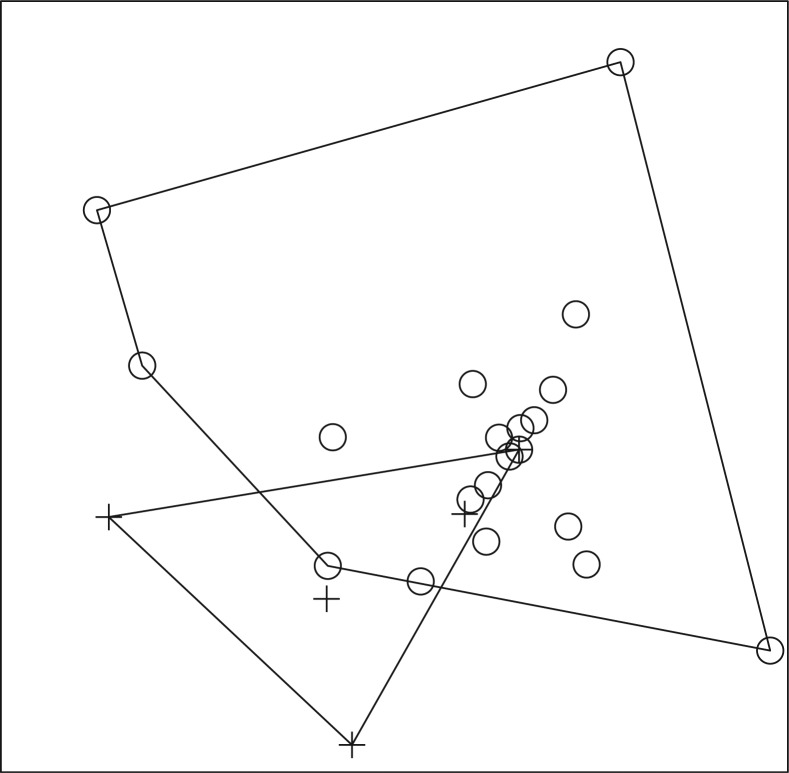
NMS ordination plot with three dimensions represented on two axes, including convex hulls, representing solution space of amphibian communities, using species presence or absence data from 2008 in relation to enoyl reductase (ER) domain positivity. Crosses represent ER-positive sites and circles represent ER-negative sites. Stress: 0.7118. Sites that cluster closest together in the ordination have the most similar community structure. Considerable overlap of sites demonstrates a lack of significant difference (ANOSIM; *P*-value=0.294) in amphibian community structure in ER-positive versus ER-negative sites in 2008.

**Fig. 5:**
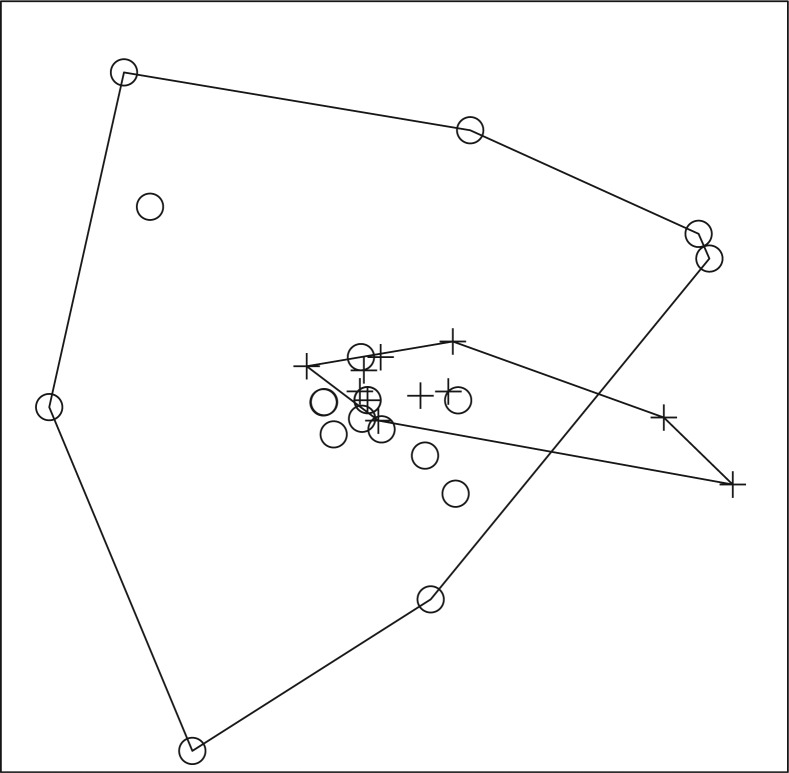
NMS ordination plot with three dimensions represented on two axes, including convex hulls, representing solution space of amphibian communities, using combined species presence or absence data from 2008 and 2009 in relation to enoyl reductase (ER) domain positivity. Crosses represent ER-positive sites and circles represent ER-negative sites. Stress: 0.6023. Sites that cluster closest together in the ordination have the most similar community structure. Considerable overlap of sites demonstrates a lack of significant difference (ANOSIM; *P*-value=0.631) in amphibian community structure in ER-positive versus ER-negative sites for combined 2008 and 2009 amphibian data.

**Fig. 6:**
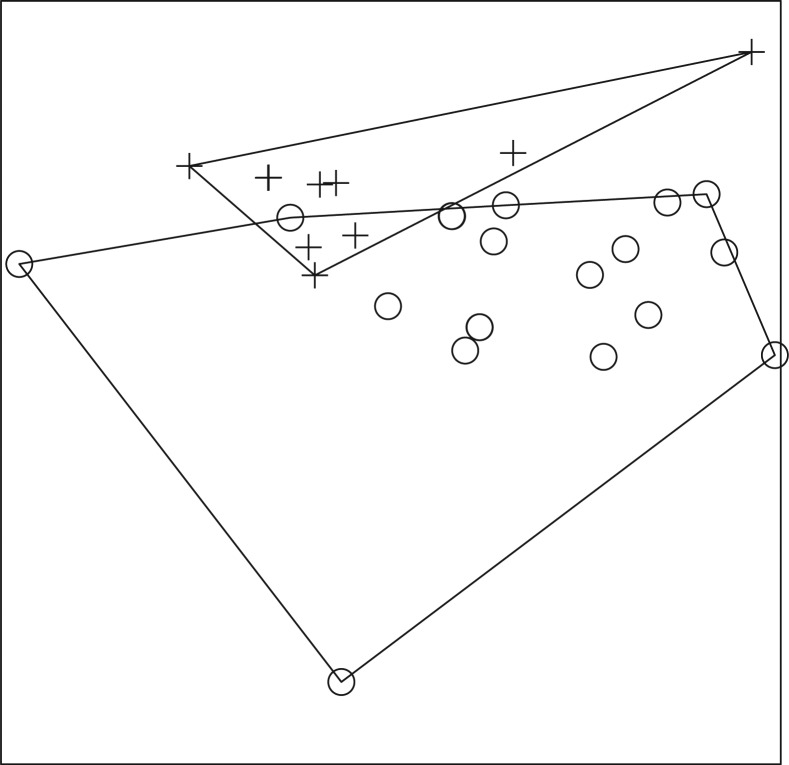
NMS ordination plot with three dimensions represented on two axes, including convex hulls, representing solution space of fish communities, using combined species presence or absence data from 2008 and 2009 in relation to enoyl reductase (ER) domain positivity. Crosses represent ER-positive sites and circles represent ER-negative sites. Stress: 0.4505. Sites that cluster closest together in the ordination have the most similar community structure. Separation of ER-positive from ER-negative sites demonstrates a significant difference (ANOSIM; *P*-value=0.031) in fish community structure in ER-positive versus ER-negative sites for combined 2008 and 2009 fish data.

**Fig. 7:**
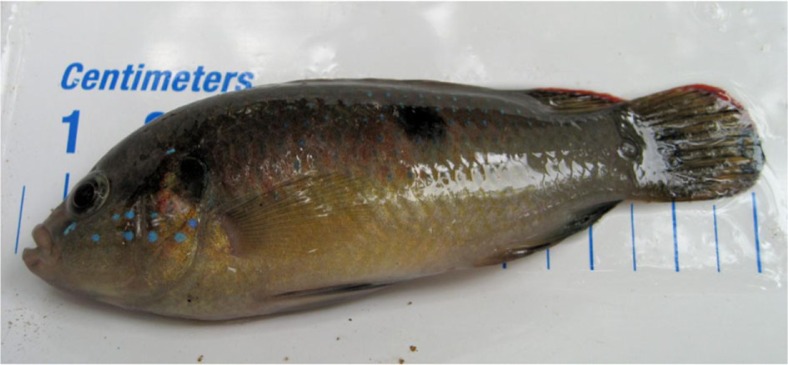
*Hemichromis bimaculatus*. This piscivorous cichlid was found at 80% of sites that tested positive for mycolactone-producing mycobacteria. A positive result was indicated by successful PCR amplification of the enoyl reductase (ER) domain, which is present in the plasmid required for mycolactone production.

A total of 587 fish, representing 13 genera and at least 17 species, and a total of 351 amphibians, representing 10 genera, were collected (Tables 1–4). In 2008, ER-positive water filters were recovered from Otinibi, Danfa, Teiman, Afiaman, and Nsakina. ER-positive specimens were found at all sites visited in 2009 (Table 5). ER positivity was not found to be associated with fish or amphibian community structure in 2008 (Figs. 3 and 4), and this was verified by ANOSIM (fish: *P*-value=0.672; amphibian: *P*-value=0.294). Combined amphibian data from 2008 and 2009 also failed to show a significant association with ER positivity (*P*-value=0.631; Fig. 5). However, when fish community data from 2008 and 2009 were combined, there was a significant difference in fish communities between ER-positive and ER-negative sites (*P*-value=0.031; Fig. 6). Weija was eliminated from these analyses because the different sampling protocol for that site did not allow for collection of community data. A SIMPER analysis on combined fish data showed that *Hemichromis bimaculatus*, *Sarotherodon* sp., and *Barbus sublineatus* were responsible for 47% of the dissimilarity between fish communities in ER-positive and ER-negative sites. *H*. *bimaculatus* (Fig. 7) was the taxon responsible for the most dissimilarity (22%) and was present in 80% of ER-positive sites versus 11% of ER-negative sites. However, *H*. *bimaculatus* specimens did not show a significant difference in ER positivity when compared to other fish species (*P*-value=0.771).

**Table 1:** Amphibian taxa collected in 2008

Site	MPM	AF	AM	BU	CH	HE	HY	KA	LE	Unk
Otinibi	Pos						1			3
Danfa	Pos									
Teiman	Pos									
Afiaman	Pos									
Kotoku	Neg	11					6			
Nsakina	Pos	13					1			
Pampamwie	Neg								6	
Ata Kofi	Neg	5	6				9			
Titiaka	Neg		3	2					4	
Wawaso	Neg									
Asato	Neg		31							
Okaniase	Neg									
Adaklu Dzakpo	Neg									
Adaklu Amedzivie	Neg	1					10			
Adaklu Dorkpo	Neg	5					1			
Agodeke	Neg									
Abutia Kloe	Neg	6				1	6	1		
Wayanu	Neg			1			2			
Laweh Kope	Neg									
Asutuare Junction	Neg						4			
Asebi	Neg									
Dedenya	Neg						22			
Mensah Bar	Neg									
Saduase	Neg	1			1		15			
Oyibi	Neg									

Sites were classified according to presence of mycolactone-producing mycobacteria (MPM), which was determined by subjecting water filter samples to PCR targeting the enoyl reductase (ER) domain present in the plasmid required for mycolactone production.

AF, *Afrixalus* sp.; AM, *Amnirana* sp.; BU, *Bufo* sp.; CH, *Chiromantis* sp.; HE, *Hemisus* sp.; HY, *Hyperolius* sp.; KA, *Kassina* sp.; LE, *Leptopelis* sp.; Unk, unidentified.

**Table 2:** Amphibian taxa and mycolactone-producing mycobacteria (MPM) presence for sites visited in 2009

Site	MPM	AF	BU	HE	HY	LE	RA	XE	Unk
Djorse	Pos	12	10				4		
Otuaplem	Pos	4		10		1	2	12	
Sarpeiman	Pos	1	13		8				
Seduase	Pos	9							
Achiaman	Pos				18		1		
Kwashikuma	Pos	16			48		3		1
Weija	Pos								

MPM presence was determined by subjecting fish and amphibian tissue samples to PCR targeting the enoyl reductase (ER) domain present in the plasmid required for mycolactone production.

AF, *Afrixalus* sp.; BU, *Bufo* sp.; HE, *Hemisus* sp.; HY, *Hyperolius* sp.; LE, *Leptopelis* sp.; RA, Ranidae; XE, *Xenopus* sp.; Unk, unidentified.

**Table 3:** Fish taxa collected in 2008

Site	MPM	AW	BB	BM	BP	BS	BT	CY	FT	HB	HF	PA	PO	PS	SA	SY
Otinibi	Pos					19		1							1	
Danfa	Pos					6				8					6	
Teiman	Pos									3			1		21	
Afiaman	Pos		1							1	1			1	1	
Kotoku	Neg														5	
Nsakina	Pos													1		
Pampamwie	Neg				6	25										
Ata Kofi	Neg				9	26										
Titiaka	Neg						1								1	
Wawaso	Neg			1		9										
Asato	Neg							3								
Okaniase	Neg					9										
Adaklu Dzakpo	Neg			25			11								1	
Adaklu Amedzivie	Neg						19		7							
Adaklu Dorkpo	Neg	10		7	27										4	
Agodeke	Neg				4		26		15			1				
Abutia Kloe	Neg															
Wayanu	Neg				2	11										2
Laweh Kope	Neg		2	30							1				7	
Asutuare Junction	Neg			28						4					10	
Asebi	Neg			41							1				11	
Dedenya	Neg							2		1					5	
Mensah Bar	Neg								9							
Saduase	Neg															
Oyibi	Neg														31	

Sites were classified according to mycolactone-producing mycobacteria (MPM) presence, which was determined by subjecting water filter samples to PCR targeting the enoyl reductase (ER) domain present in the plasmid required for mycolactone production.

AW, *Aphyosemion walker*; BB, *Brienomyrus brachyistius*; BM, *Barbus macrops*; BS, *Barbus sublineatus*; BT, *Barbus trispilos*; CY, *Cyprinodontidae*; FT, *Fundulosoma thieryi*; HB, *Hemichromis bimaculatus*; HF, *Hemichromis fasciatus*; PA, *Protopterus annectens*; PO, *Parachanna obscura*; PS, *Polypterus senegalus*; SA, *Sarotherodon* sp.; SY, *Syndontus* sp.

**Table 4:** Fish taxa and mycolactone-producing mycobacteria (MPM) presence based on results from fish and amphibian specimens for sites visited in 2009

Taxa	MPM	BS	CA	ED	HB	SA
Djorse	Pos	1		2	5	
Otuaplem	Pos		1		3	
Sarpeiman	Pos				3	
Saduase	Pos					
Achiaman	Pos		1		2	23
Kwashikuma	Pos				5	4
Weija	Pos					15

MPM presence was determined by subjecting fish and amphibian tissue samples to PCR targeting the enoyl reductase (ER) domain present in the plasmid required for mycolactone production.

BS, *Barbus sublineatus*; CA, *Clarias angularis*; ED, *Epiplatys dageti*; HB, *Hemichromis bimaculatus*; SA, *Sarotherodon* sp.

**Table 5:** Percent positive for enoyl reductase (ER) domain present in the plasmid required for mycolactone production, (total number collected) fish, tadpoles, adult frogs, and combined data for specimens collected in 2009

	Djorse	Otuaplem	Sarpeiman	Saduase	Achiaman	Kwashikuma	Weija
Fish	13 (8)	100 (4)	100 (3)	0 (0)	42 (26)	44 (9)	13 (15)
Tadpoles	41 (22)	50 (26)	73 (22)	67 (9)	47 (17)	67 (66)	0 (0)
Frogs	75 (4)	33 (3)	0 (0)	0 (0)	50 (2)	100 (2)	0 (0)
Combined	38 (34)	55 (33)	76 (25)	67 (9)	44 (45)	65 (77)	13 (15)

### DNA extraction and PCR

A subsample of extracted DNA from kidney and intestine samples was spiked with *M. ulcerans* DNA to test for PCR inhibition. High levels of inhibition were observed in intestine samples, but no evidence of inhibition was seen in kidney samples. Use of a MO BIO clean-up kit successfully removed PCR inhibition.

Of the two methods used for DNA extraction, the Käser method was 5,000 times more sensitive than the one-tube lysis method. With the one-tube procedure, the lowest detection limit using ER PCR was approximately 1,600 CFUs. With the Käser extraction procedure, the lowest detection limit was 0.32 CFUs. Addition of goldfish tissue homogenate did not decrease the detection limit, but additional non-specific binding was observed.

Negative controls consistently showed lack of contamination, and after the removal of inhibitors, positive controls consistently showed that the Käser extraction and PCR were successful. Overall, the mean ER positivity for all specimens was 54% (128/238). ER positivity was 39% (25/65) for fish, 59% (96/162) for tadpoles, and 64% (7/11) for adult frogs. Positivity rates for all specimens from a single site ranged from 13% at Lake Weija to 76% at Sarpeiman. However, Weija was removed from Fisher's exact tests due to the differing sampling method. Combined fish and amphibian counts showed a significant difference in the number of ER-positive specimens based on site (*P*-value=0.0164). As Sarpeiman showed the greatest positivity, this site was examined in a further analysis. Removing Sarpeiman (*P*-value=0.0523) indicated that this site was responsible for the significant result. A separate analysis indicated that overall, there was no difference in positivity rates between fish and amphibians (*P*-value=0.106). There was not a significant (*P*-value=1) difference in ER positivity among fish-feeding guilds (predator, insectivore, omnivore, and planktivore).

### External versus internal ER positivity

Of the 128 positive fish and amphibian specimens, 118 were identified as positive based on DNA from the intestine. Five specimens had positive results from both the intestine and the kidney, and five had positive results only from the kidney.

Twenty-seven external surface swabs were analyzed, and four of these were ER positive. Of the 27 total swabs, 14 came from fish or amphibians in which either the kidney or intestine was ER positive. Of these 14, only 1 external swab was also positive. The other three ER-positive external swabs came from fish and amphibian specimens in which the kidney and intestine were ER negative.

### VNTR

VNTR typing was attempted for all ER-positive samples, but only three of the tissue samples and no swab samples were successfully VNTR typed. Two were determined to be *M. liflandii*, a mycolactone-producing mycobacterium pathogenic to frogs. These were recovered from the intestine of a *Hyperolius* tadpole from Kwashikuma and the intestine of a predatory cichlid, *H. bimaculatus* from Djorse. One specimen, an adult frog from the genus *Leptopelis*, collected at Otuaplem, was confirmed to be positive for *M. ulcerans*. This is the first known finding of *M. ulcerans* in an adult amphibian. VNTR typing of water filters collected in 2008 confirmed *M. ulcerans* presence on filters from Otinibi and Nsakina and the presence of other mycolactone-producing mycobacteria on filters from Teiman. Filters from all other sites visited in 2008 were ER-negative, and therefore were not VNTR typed.

## Discussion

Site ER positivity was not statistically associated with specific fish or amphibian communities and showed no association between any certain species and ER positivity in 2008, but when 2009 data were included, there was a significant association between the predatory cichlid *H. bimaculatus* and site ER positivity. It is likely that this became evident only when the 2009 data were included because of the low number of ER-positive sites visited in 2008. Because *H. bimaculatus* specimens did not show higher positivity rates than other fish specimens, it is unlikely that the site positivity is a result of the presence of *H. bimaculatus*. Rather, the presence of *H. bimaculatus* may indicate a suitable environment for mycolactone-producing mycobacteria. While *H. bimaculatus* is widespread in West Africa, its preferred habitats include forested and recently disturbed areas, and sand or mud-bottomed water bodies (43). It should be noted that this sampling scheme was qualitative, representing a single point in time. To fully characterize fish and amphibian communities, more intensive and extensive sampling would be required.

Positivity of fish communities varied among sites, which may suggest that the environmental distribution of *M. ulcerans* is a part of the ecology of highly focal water bodies. However, it may also be an artifact related to small sample sizes in those locations. Fish appear likely to come into contact with the bacterium regardless of feeding guild. In addition, while it is not apparent that fish act as replicative reservoirs for mycolactone-producing mycobacteria, including *M. ulcerans*, they do accumulate bacteria at levels detectable by standard PCR and offer a potential tool for environmental screening. Feeding-guild analyses were not performed for amphibians because little is known about the specific feeding methods of tadpoles and because all adult frogs are predators. However, neither genus nor site was significantly linked to positive ER status among amphibians.

The overall fish ER positivity rate (39%) was higher in this study than IS*2404* positivity rates reported in previous studies (10 and 20%, respectively) (12, 28). This was unexpected considering the much higher sensitivity of IS*2404* PCR. Possible reasons for the difference include annual and seasonal variation in positivity rates, increased sample sizes in the current study, and differing extraction methods. For example, the one-tube extraction method was attempted in the current study, but the Käser extraction method was chosen because it resulted in detection limits that were over 5,000 times more sensitive. Site was shown to be an important factor in the current study, so number and location of sites could also explain the difference. Contamination was unlikely a factor since controls consistently showed a lack of contamination. Nearly all kidney samples were negative, providing further evidence that contamination was not a factor. The possibility of PCR inhibition in previous studies must be considered, especially given the high amount of inhibition initially observed in the present study.

Amphibians did not have a higher rate of ER positivity than fish. For amphibians, it is important to consider the slow rate of development of *M. ulcerans* and the transience of the aquatic tadpole stage. Timing of tadpole development is highly variable, depending on species, temperature, food availability, and density (36). *M. ulcerans* or other mycolactone-producing mycobacteria acquired by tadpoles with very short aquatic stages would potentially have very little time to replicate while the amphibian remained in the tadpole stage, and might not be detectable until after the frog has gone through metamorphosis. For this reason, adult frogs may be better targets than tadpoles for future studies. The single specimen where *M. ulcerans* was verified by VNTR was an adult frog, though *M*. *liflandii* was found in a tadpole. This is the first report of *M. ulcerans* in a terrestrial amphibian, and may represent a potential aquatic-terrestrial linkage, as well as a potential dispersal method for the pathogen. In a study performed in Brazil, *Mycobacteria chelonei* and *M. fortuitum*, which are opportunistic mycobacterial pathogens of humans, were shown to survive digestion by adult amphibians, were excreted through the feces for 7–10 days after being consumed, and were found in soil and water samples taken from amphibian-breeding grounds (44). Similar studies on *M. ulcerans* would be useful to determine whether or not *M. ulcerans* may be disseminated by a similar method. In addition to potentially spreading *M. ulcerans* throughout breeding areas via fecal material, it is possible that amphibians may pass the bacterium to terrestrial predators. To our knowledge, there are no known cases of Buruli ulcer disease that have been linked to handling amphibians; however, the closely related *M. marinum* can be directly transmitted to humans from fish, usually during tending of home aquaria (45, 46). While *M. ulcerans* has traditionally been associated with aquatic habitats, it has recently been found in terrestrial mammals in Australia (14). Thus, this first report of a terrestrial vertebrate with *M. ulcerans* in Africa suggests future research should include organisms with an aquatic-terrestrial linkage.

The majority of positive ER results came from intestine samples of both fish and frogs. External swabs had an overall ER positivity rate of only 15%, suggesting that while bacteria may sometimes adhere to the external surface of an organism, ER-positive intestine results were actually due to bacteria in the intestine and not due to external contamination of samples. The very low rate of ER-positive kidney samples suggests that bacteria were acquired during feeding and were passing through the intestine without causing a systematic infection. If the bacteria were acquired through injection, such as during attack by predators, they are likely be detected in the kidney as well (47).

Despite finding relatively high ER positivity rates in fish and amphibians, very few samples were successfully VNTR typed. This is likely due to the low proportions of *M. ulcerans* or other mycolactone-producing mycobacterial DNA in the samples. DNA was further depleted by early extraction issues and repeated PCRs. Many of the ER bands were very faint, consistent with small quantities of DNA. VNTR amplification is less sensitive than ER-PCR, so it is likely that DNA could be detected by ER amplification but not by VNTR amplification. Previous authors have reported low sensitivity for VNTR of environmental samples, with over 100 genomes µL^−1^ DNA or at least 10^5^ organisms/gram required for successful VNTR analysis (14, 33). Low quantities of DNA in fish are consistent with the conclusion of previous authors that *M. ulcerans* does not replicate in fish (47). For fish, it appears that *M. ulcerans* and other mycolactone-producing mycobacteria are likely encountered during feeding, before passing through the digestive tract. This could be further investigated by testing fecal material separately from intestinal tissue.

The relatively high levels of PCR inhibition experienced during the course of this study reinforce the need to consider inhibition when dealing with environmental samples. Numerous extraction methods have been used to test for *M. ulcerans* (31, 32, 39, 48, 49). The Käser method was chosen for this study specifically because it was a recently developed, optimized combination of well-known methods; was developed with testing of environmental samples in consideration; was relatively inexpensive; and was validated with insect samples. However, despite working well on control fish tissue, the method did not remove environmental inhibitors from actual field samples. Other studies have also found problems with inhibition (49, 50). There are numerous methods for removing environmental inhibitors, including the MO BIO kit used in this study. However, because the methods vary in effectiveness, there is a need for a standardized extraction method for use in *M. ulcerans* studies to be able to meaningfully compare results.

This study represents the first VNTR confirmation of *M. ulcerans* or *M. liflandii* in wild amphibian and fish populations in West Africa. Results from this study suggest that adult amphibians should be carefully examined as potential reservoirs for *M. ulcerans* in West Africa, and that *H. bimaculatus* may be useful as an indicator of habitats likely to support mycolactone-producing mycobacteria. These data support a feeding linkage within aquatic habitats considering that *M. ulcerans* has been detected in biofilms on aquatic plants as well as in many different aquatic invertebrate taxa (8, 31). In addition, since *M. ulcerans* was typed from an adult frog, these results suggest that *M. ulcerans* is both part of the aquatic food web and the terrestrial food web. In light of the findings of previous investigators regarding the passage of mycobacteria through adult frogs (44), contact with areas that contain high densities of adult frogs may provide a terrestrial means of exposure of humans to *M. ulcerans*.
